# Combined Conduit Score in Contrast-Enhanced Magnetic Resonance Venography in Patients with Idiopathic Intracranial Hypertension: Neuro-interventionalistsʼ vs. Radiologistsʼ Assessment

**DOI:** 10.1007/s00062-023-01263-5

**Published:** 2023-02-17

**Authors:** Nada Elsaid, Ahmed Razek, Nihal M. Batouty, Ali H Elmokadem, Ahmed M. Tawfik, Ahmed Saied

**Affiliations:** 1 Department of Neurology, Faculty of Medicine, Mansoura University, Mansoura, Egypt; 2 Department of Diagnostic and interventional Radiology, Faculty of Medicine, Mansoura University, Mansoura, Egypt

**Keywords:** Transverse sinus, Stenosis, Stenting, Pseudotumor cerebri, MRV

## Abstract

**Background:**

Based on increased understanding of the idiopathic intracranial hypertension (IIH) pathophysiology, venous sinus stenting (VSS) has emerged as an effective treatment for patients with transverse sinus stenosis (TSS). The presence of a reliable TSS screening tool is warranted. The combined conduit score (CCS) is the most widely used score for evaluation of the cerebral sinovenous stenosis in contrast-enhanced magnetic resonance venography (CE-MRV).

**Purpose of the Study:**

To evaluate the interobserver agreement between neuro-interventionalists and radiologists with respect to the CCS in evaluation of transverse sinus stenosis in patients with idiopathic intracranial hypertension using CE-MRV.

**Methods:**

A retrospective study was conducted on 26 consecutive patients diagnosed with IIH and underwent CE-MRV. The 2 neuro-interventionalists and 2 radiologists separately evaluated the cerebral venous sinuses using the CCS.

**Results:**

The mean CCS was significantly different between the neuro-interventionalists and radiologists (*p* < 0.001), higher for the radiologists. The inter-rater reliability was excellent (ICC = 0.954, 95% CI: 0.898–0.979) between the 2 neuro-interventionalists, good between the 2 radiologists (ICC = 0.805, 95% CI: 0.418–0.921), but was not acceptable between the neuro-interventionalists and the radiologists (ICC 0.47 95% CI:−2.2–0.782).

**Conclusion:**

Despite the excellent agreement between the neuro-interventionists and the good agreement between the radiologists, there was no agreement between the neuro-interventionists and the radiologists. Our finding suggests that there is a gap between the 2 specialties but does not favor any of them. Factors related to the observers, the venous sinus system, the MRV or the CCS score may have resulted in this discrepancy. Automatic or semi-automatic feature extractions to produce quantifiable biomarkers for IIH are warranted. The clinical decisions should not depend only on strongly observer-dependent scores with training and/or experience-dependent influences.

## Introduction

Idiopathic intracranial hypertension (IIH) or more recently chronic intracranial venous hypertension syndrome (CIVHS) is a challenging condition to treat. Recently, the role of raised intracranial venous pressure in the pathophysiology of IIH has been emerging. The in-depth understanding of this pathophysiology led to the introduction of venous sinus stenting (VSS) as a safe and effective therapeutic option for some IIH patients. Stratifying the IIH patients according to venous outflow status can help in the selection of patients who are more likely to respond to VSS [1–3].

Transverse sinus stenosis (TSS) is strongly associated with IIH. The presence of a reliable transverse sinus stenosis (TSS) screening tool is warranted. The combined conduit score (CCS) is the most widely used score for evaluation of the cerebral sinovenous stenosis in contrast-enhanced magnetic resonance cerebral venography (CE-MRV). It was first introduced by Farb et al. in 2003 and have been well recognized since then as highly sensitive and specific predictive tool for IIH [4, 5].

This score was reported to have excellent interobserver agreement by Farb et al. but to our knowledge, there are no other studies that focused on the interobserver variability of this score, and for sure we are the first to compare between the neuro-interventionalistsʼ and radiologistsʼ evaluation of CCS in CE-MRV [4].

## Methods

### Study Population

*This is a Retrospective Study.* Mansoura Faculty of Medicine Institutional Research Board (MFM-IRB) approval was obtained (R.22.09.1826). Data were obtained from 26 patients diagnosed with IIH after fulfilling the modified Dandy criteria and undergoing CEMRV [6].

### MR Acquisition

The MRV was performed using 2D time of flight (TOF) MRV followed by contrast-enhanced MRV on a 1.5 T 16-channel MR scanner (GE Healthcare H.D., Milwaukee, WI, USA). The parameters used for 2D TOF MRV sequence were as follows: TR/TE, 18/3.1 ms; flip angle (FA), 20; 40; section thickness, 1.4 mm; FOV, 220 mm; acquisition matrix, 256 × 160 and acquisition in coronal image plane.

The post-contrast MRV study was done using the time-resolved imaging of contrast kinetics (TRICKS) technique with the following parameters: TR/TE, 36/1.4 ms; FA, 30; section thickness, 1.4 mm; acquisition matrix, 256 × 160; TA, 2 min and acquisition in coronal image plane.

Ten ml contrast agent (gadolinium-based) was injected intravenously at a rate of 2 ml/s using an injector, followed by a 20 ml saline flush.

### Image Analysis

**Fig. 1:**
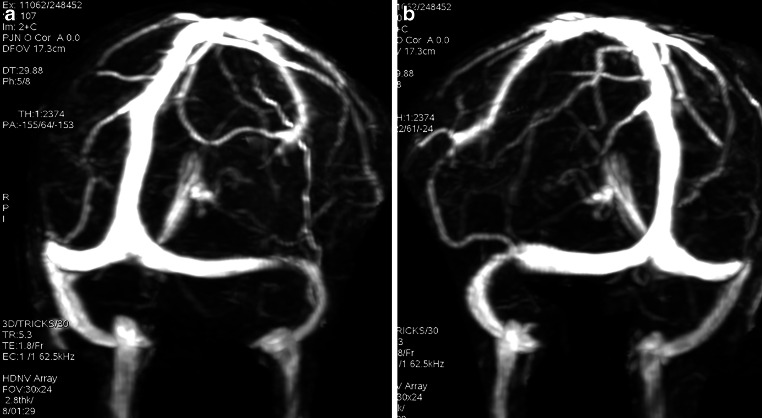
Post-contrast magnetic resonance venography images of the cerebral veins of a 36-year-old female idiopathic intracranial hypertension patient (maximum intensity projection, MIP, images, left posterior oblique view (**a**), right posterior oblique view (**b**). The combined conduit score for the first neuro-interventionalist was 3, for the second neuro-interventionalist was 2, for the first radiologist was 5 and for the second radiologist was 8

The axial CE-MRV source images and the same standard static views and clips for the 3D reconstruction images were reviewed on the picture archiving and communication system (PACS) system by 2 neuro-interventionalists (AS 13 years experience and NE 10 years experience) and 2 radiologists with a special background in neuroradiology or vascular imaging (NB 16 years experience and AT 19 years experience) blinded to each others results to assign a CCS. Then, as a reference for each group a consensus reading was obtained. Each of the transverse and sigmoid sinuses was scored separately from 0 to 4 based on the maximum degree of stenosis from the confluence to the end of the sigmoid sinus. The presence of discontinuation or flow gap or aplastic segment was score 0; hypoplasia or severe segmental stenosis in reference to the distal superior sagittal sinus (SSS) (< 25% of the SSS diameter) was scored 1; moderate segmental sinus stenosis (25–50%) was scored 2, mild segmental sinus stenosis (50–75%) was scored 3 and absence of significant narrowing (75–100%) was scored 4. The scores of both sinuses were added to create the CCS (Fig. 1; [4]).

## Statistical Analysis

Data were analyzed using the statistical package for the social sciences (SPSS) software package version 20.0 (IBM Inc., Armonk, NY, USA). Normal data distribution was confirmed using the Kolmogorov Smirnov test. Parametric data are expressed in mean ± standard deviation. Non-parametric data are expressed in median and range. Numbers and percentages are used to describe qualitative data. A *P*-value < 0.05 was considered to be significant. Interclass correlation coefficient (ICC) was used to measure the interobserver agreement between each reader and the between the consensus readings. An ICC value of 1.0 represents a perfect agreement, 0.81–1.0 is an excellent agreement, 0.61–0.80 is a good agreement, 0.41–0.6 is moderate agreement, < 0.41 is poor agreement. Pearson correlation was used to measure the correlation between the cerebrospinal fluid (CSF) opening pressure and the different CCS readings.

## Results

### Clinical Data

The study participants were 23 females and 3 males with mean age of 32 ± 2.6 years. The mean cerebrospinal fluid opening pressure was 41.6 ± 9 mmH_2_O. There was no significant correlation between the CSF opening pressure and any of the different CCS readings.

### Inter-neuro-interventionalists Agreement

**Fig. 2:**
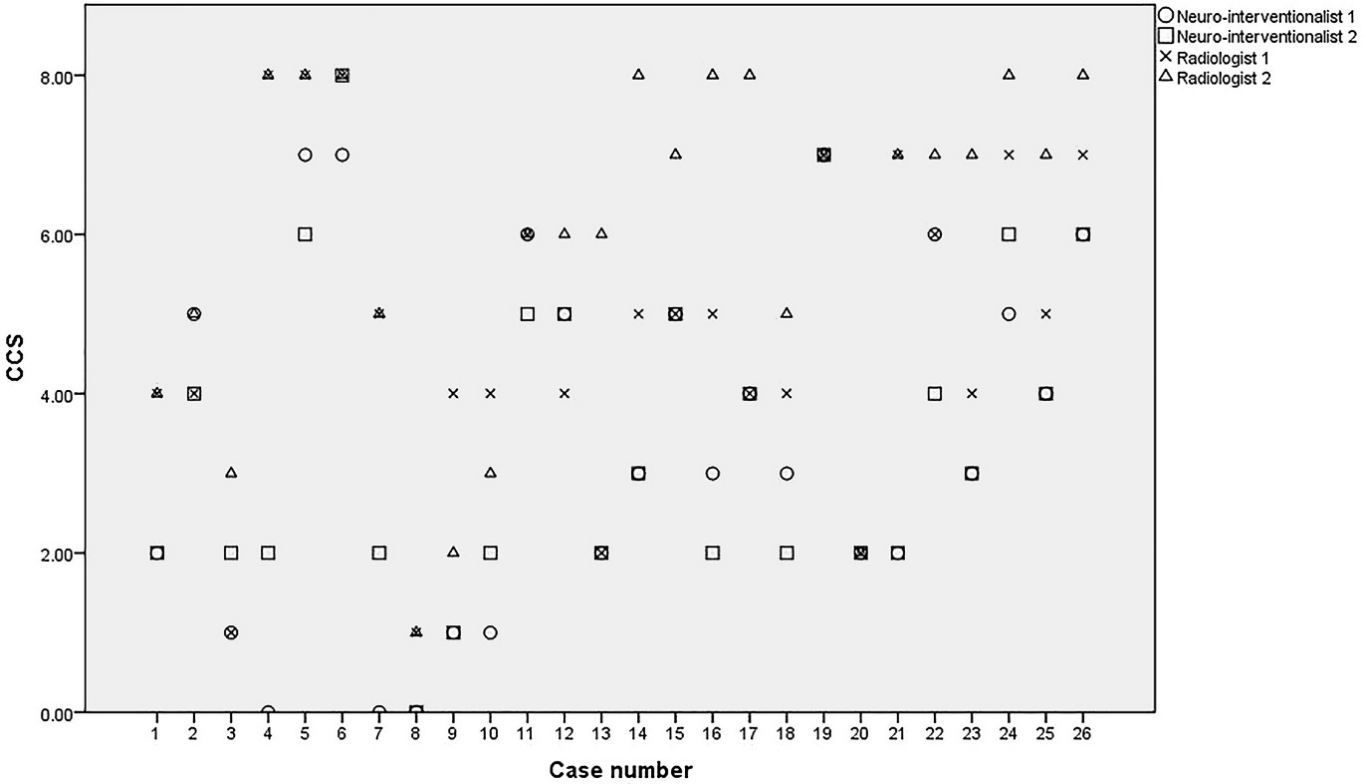
Scatter plot presenting the determined combined conduit scores (CCS) for each reader across the study subjects

The mean deduced CCS was 3.46 for the first neuro-interventionalist and 3.5 for the second. Inter-rater reliability was excellent (ICC = 0.954, 95% CI: 0.898–0.979) (Table 1; Fig. 2).

**Table 1:** Inter-neuro-interventionalists agreement

	1st Neuro-interventionalists	2nd Neuro-interventionalists	Intraclass correlation	95% Confidence interval	
				Lower bound	Upper bound
Average CCS	3.46 ± 2.28	3.5 ± 2	0.954	0.898	0.979

*CCS* Combined conduit score

### Inter-radiologists Agreement

The mean deduced CCS was 4.88 for the first radiologist and 5.9 for the second. Inter-rater reliability was good (ICC = 0.805, 95% CI: 0.418–0.921) (Table 2; Fig. 2).

**Table 2:** Inter-radiologists agreement

	First radiologist	Second radiologist	Intraclass correlation	95% Confidence interval	
				Lower bound	Upper bound
Average CCS	4.88 ± 2	5.92 ± 2.18	0.802	0.418	0.921

*CCS* combined conduit score

### Inter-neuro-interventionalists and Radiologists Agreement

Inter-rater reliability was inconsistent between each of the neuro-interventionalists compared to each radiologist (Table 3). As regards the approved consensus between the 2 neuro-interventionalists and between the 2 radiologists the mean CCS was significantly lower in the neuro-interventionalistsʼ consensus (3.38 ± 2.22) in comparison to the radiologistsʼ consensus (5.9 ± 1.9) (*p* < 0.001). Inter-rater reliability was not acceptable between the neuro-interventionalists and radiologists consensus (ICC 0.47 95% CI:−2.2–0.782) (Table 3).

**Table 3:** Inter-neuro-interventionalists and radiologists agreement

	First radiologist	Second radiologist	Radiologists consensus
1st Neuro-interventionalist	ICC 0.63 95% CI 0.1–0.84	ICC 0.54 95% CI −2.1–0.82	N/A
2nd Neuro-interventionalist	ICC 0.69 95% CI 0.11–0.88	ICC 0.55 95% CI −2.3–0.84	N/A
Neuro-interventionalists consensus	N/A	N/A	ICC 0.47 95% CI −2.2–0.782

*ICC* Inter class correlation, *CI* confidence interval, *N/A* not applicable

## Discussion

The utilization of VSS as a safe, effective and definitive treatment for the refractory IIH cases with bilateral TSS severe enough to compromise cerebral venous outflow is rapidly rising [1, 2, 7–10].

This necessitates the presence of a reliable tool for diagnosis of the venous sinus stenosis in such patients. MR venography (MRV) is the most used modality for identification of venous sinus pathology in patients of IIH [4]. It can detect transverse sinus stenosis, which is reported to be present in about 90% of IIH patients. Several scores and indices have been proposed for evaluation of the sinovenous system; however the CCS is the most widely used [4, 11].

The aim of our study was to evaluate the interobserver agreement between neuro-interventionalists and radiologists, as regards the CCS in evaluation of TSS in patients of IIH using CE-MRV.

Our results showed that there was an excellent agreement between the 2 neuro-interventionalists (ICC = 0.954, 95% CI: 0.898–0.979), and good agreement between the 2 radiologists (ICC = 0.805, 95% CI: 0.418–0.921). This is in line with the excellent agreement level (0.9) previously reported by Farb et al., who proposed the score in the first place [4]. To our knowledge there are no reports for external examination of the interobserver agreement of this score.

Despite the previously mentioned agreement, the mean CCS was significantly different between the neuro-interventionalists and radiologists (*p* 0.0000001), higher for the radiologists, and the inter-rater reliability was not acceptable (ICC 0.47, 95% CI: −2.2–0.782).

Our results suggest that in comparison to each other, neuro-interventionalists may tend to overestimate the lesion, while radiologists may tend to underestimate it. Being the first to compare the agreement between the neuro-interventionalists and radiologists, it is difficult to explain these results; however, several proposed factors may have led to this discrepancy. These factors may be related to the observers, the venous sinus system, the MRV or the CCS score.

### Observer-related Factors

Being exposed all the time to medical refractory cases of IIH, digital subtraction cerebral venography images, venous sinus pressures and the associated gradients, neuro-interventionalists tend to be preoccupied by the degree of the stenosis and the severity that may compromise the cerebral venous outflow and the CSF drainage [12].

This is supported by the fact that reproducibility generally depends on the different learning curves of the examiners and prior individual experience and training [11].

### Venous Sinus System-related Factors

Various venous variations, including focal stenosis, hypoplasia, arachnoid granulations, fenestrated sinuses and fibrous septa have been reported to be implicated in the etiology of IIH syndrome [4, 13–16].

Confusingly, unilateral transverse sinus reduced diameter is reported to be present in about 30% of normal individuals without raised intracranial pressure [17]. Additionally, dural venous sinus stenosis can be classified into intrinsic and extrinsic stenosis. Intrinsic stenosis is in cases of intraluminal filling defects such as arachnoid granulations or thrombi. Extrinsic stenosis is in cases of extraluminal compression on the sinus by the surrounding brain parenchyma [18].

These factors may affect the judgment of the readers according to their level of exposure to them, which is higher for the neuro-interventionalists as the nature of transverse sinus affection may seem nonspecific in the MRV sequence especially for the following entities: anatomic variations, sinus asymmetry, sinus indentations due to arachnoid granulations or previous dural sinus thrombosis sequelae [5].

### MRV-related Factors

In the MRV technique the transverse and sigmoid sinuses are locations routinely affected by signal loss due to artifacts that occurs as result of in-plane flow and turbulence. This is one of the well-documented limitations of MRV, however, less common with CE-MRV. Being more known to the radiologists, the radiologists may tend to underestimate the lesions and overlook them as artifacts [4].

### CCS Score-related Factors

The CCS as a score may have some pitfalls. CCS adopts the utilization of 25% as the level of change between the 4 proposed scales; however, da Silveira Carvalho et al. thought that is not as fast or easy as the use of one third [11].

In reference to the CCS, hypoplasia was defined together with severe stenosis as < 25% segmental reduction of the sinus diameter; however, hypoplasia was defined by others as 1/3 reduction in the full length of the transverse sinus diameter rather than a segment [11]. This may create confusion for the readers.

Also, CCS uses the distal SSS as the comparative reference for evaluation of the degree of the stenosis. This may be challenging due to the possible poor visualization of the SSS in the MRV due to occurrence of MR flow artifacts [19]. Others proposed a comparison with the immediate prestenosis segment rather than the SSS [11].

Finally, the CCS does not warrant differentiation between the site of stenosis either transverse or sigmoid. This is one of the proposed limitations of the CCS especially that some regions are more prone to artifacts than others [4].

### Limitations and Recommendations

Our study has several limitations. First the sample size is relatively small. Second, the study was not blinded as the study included only IIH patients. Further large sampled blinded randomized controlled studies are needed. Third, it is unclear if the more pathological scores by the interventionalists reflect expertise or observer-related bias. This can be confirmed or denied only by further research with reference to digital subtraction venography and the measured venous sinus pressures and gradients.

## Conclusion

Despite the excellent agreement between the neuro-interventionists and the good agreement between the radiologists, there was no agreement between the neuro-interventionists and the radiologists. Our finding suggests that there is a gap between the specialties but does not favor any. Factors related to the observers, the venous sinus system, the MRV or the CCS score may have resulted in this discrepancy. Automatic or semi-automatic feature extractions to produce quantifiable biomarkers for IIH are warranted. The clinical decisions should not depend only on strongly observer-dependent scores with training and/or experience-dependent influences.

## References

[1] Fargen KM (2020). Idiopathic intracranial hypertension is not idiopathic: proposal for a new nomenclature and patient classification. J Neurointerv Surg.

[2] Fargen KM (2021). A unifying theory explaining venous sinus stenosis and recurrent stenosis following venous sinus stenting in patients with idiopathic intracranial hypertension. J Neurointerv Surg.

[3] Townsend RK, Fargen KM (2021). Intracranial venous hypertension and venous sinus stenting in the modern management of idiopathic intracranial hypertension. Life.

[4] Farb RI, Vanek I, Scott JN, Mikulis DJ, Willinsky RA, Tomlinson G (2003). Idiopathic intracranial hypertension: the prevalence and morphology of sinovenous stenosis. Neurology.

[5] Morris PP, Black DF, Port J, Campeau N (2017). Transverse sinus stenosis is the most sensitive MR imaging correlate of idiopathic intracranial hypertension. AJNR Am J Neuroradiol.

[6] Wall M, Kupersmith MJ, Kieburtz KD, Corbett JJ, Feldon SE, Friedman DI, Katz DM, Keltner JL, Schron EB, McDermott MP, NORDIC Idiopathic Intracranial Hypertension Study Group (2014). The idiopathic intracranial hypertension treatment trial: clinical profile at baseline. Jama Neurol.

[7] Reid K, Winters HS, Ang T, Parker GD, Halmagyi GM (2022). Transverse sinus stenting reverses medically refractory idiopathic intracranial hypertension. Front Ophthalmol.

[8] Cappuzzo JM, Hess RM, Morrison JF, Davies JM, Snyder KV, Levy EI, Siddiqui AH (2018). Transverse venous stenting for the treatment of idiopathic intracranial hypertension, or pseudotumor cerebri. Neurosurg Focus.

[9] Daggubati LC, Liu KC (2019). Intracranial venous sinus stenting: a&nbsp;review of idiopathic intracranial hypertension and expanding indications. Cureus.

[10] Al-Mufti F, Dodson V, Amuluru K, Walia J, Wajswol E, Nuoman R, Keller IA, Schonfeld S, Roychowdhury S, Gupta G (2019). Neuroendovascular cerebral sinus stenting in idiopathic intracranial hypertension. Intervent Neurol.

[11] da Silveira Carvalho GB, de Andrade MSL, Idagawa MH, Tibana LA, De Carvalho RS, Silva ML, Cogo-Moreira H, Jackowski AP, Abdala N (2017). A new index for the assessment of transverse sinus stenosis for diagnosing idiopathic intracranial hypertension. J Neurointerv Surg.

[12] Fargen KM, Wolfe SQ, Traunero JR, Iyer AM, Kittel C (2022). A descriptive study of venous pressures and gradients while awake and both pre-and post-stent under anesthesia in patients with idiopathic intracranial hypertension. J Neurointerv Surg.

[13] Durst CR, Ornan DA, Reardon MA, Mehndiratta P, Mukherjee S, Starke RM, Wintermark M, Evans A, Jensen ME, Crowley RW, Gaughen J. Prevalence of dural venous sinus stenosis and hypoplasia in a generalized population. J Neurointerv Surg. 2016;8:1173–7. 10.1136/neurintsurg-2015-012147. 10.1136/neurintsurg-2015-012147 26747875

[14] Liang L, Korogi Y, Sugahara T, Ikushima I, Shigematsu Y, Takahashi M, Provenzale JM (2016). Normal structures in the intracranial dural sinuses: Delineation with 3D contrast-enhanced magnetization prepared rapid acquisition Gradient-Echo imaging sequence. Am J Neuroradiol.

[15] Battal B, Hamcan S, Akgun V, Sari S, Oz O, Tasar M, Castillo M (2016). Brain herniations into the dural venous sinus or calvarium: MRI findings, possible causes and clinical significance. Eur Radiol.

[16] Tian Y, Zhang Z, Jing J, Dong K, Mo D, Wang Y (2021). Anatomic variation of the lateral Sinus in patients with idiopathic intracranial hypertension: delineation with black-blood contrast-enhanced MRI. Front Neurol.

[17] Bono F, Lupo MR, Lavano A, Mangone L, Fera F, Pardatscher K, Quattrone A (2003). Cerebral MR venography of transverse sinuses in subjects with normal CSF pressure. Neurology.

[18] Sundararajan SH, Ramos AD, Kishore V, Michael M, Doustaly R, DeRusso F, Patsalides A (2021). Dural venous sinus stenosis: why distinguishing intrinsic-versus-extrinsic stenosis matters. Am J Neuroradiol.

[19] Ayanzen RH, Bird CR, Keller PJ, McCully FJ, Theobald MR, Heiserman JE (2000). Cerebral MR venography: normal anatomy and potential diagnostic pitfalls. Am J Neuroradiol.

